# Quantitative dynamic contrast-enhance MRI parameters for rectal carcinoma characterization: correlation with tumor tissue composition

**DOI:** 10.1186/s12957-023-03193-5

**Published:** 2023-09-26

**Authors:** Jie Yuan, Kun Liu, Yun Zhang, Yuchan Yang, Huihui Xu, Gang Han, Hua Lyu, Mengxiao Liu, Wenli Tan, Zhen Feng, Hangjun Gong, Songhua Zhan

**Affiliations:** 1 Department of Radiology, Shuguang Hospital Affiliated to Shanghai University of Traditional Chinese Medicine, Shanghai, 201203 China; 2 Department of Pathology, Shuguang Hospital Affiliated to Shanghai University of Traditional Chinese Medicine, Shanghai, 201203 China; 3 Department of Gastrointestinal Surgery, Shuguang Hospital Affiliated to Shanghai University of Traditional Chinese Medicine, Shanghai, 201203 China; 4 Department of Science and Technology, Shuguang Hospital Affiliated to Shanghai University of Traditional Chinese Medicine, Shanghai, 201203 China; 5 Diagnostic Imaging, MR Scientific Marketing, Siemens Healthineers Ltd., Shanghai, 201203 China

**Keywords:** Magnetic resonance imaging, Dynamic contrast-enhanced rectal carcinoma, Tumor tissue composition

## Abstract

**Objective:**

To investigate the relationship between dynamic contrast-enhanced (DCE) magnetic resonance imaging (MRI) measurements and the potential composition of rectal carcinoma.

**Methods:**

Twenty-four patients provided informed consent for this study. DCE-MRI was performed before total mesorectal excision. Quantitative parameters were calculated based on a modified Tofts model. Whole-mount immunohistochemistry and Masson staining sections were generated and digitized at histological resolution. The percentage of tissue components area was measured. Pearson correlation analysis was used to evaluate the correlations between pathological parameters and DCE-MRI parameters.

**Results:**

On the World Health Organization (WHO) grading scale, there were significant differences in extracellular extravascular space (K^trans^) (*F* = 9.890, *P* = 0.001), mean transit time (MTT) (*F* = 9.890, *P* = 0.038), CDX-2 (*F* = 4.935, *P* = 0.018), and Ki-67 (*F* = 4.131, *P* = 0.031) among G1, G2, and G3. ECV showed significant differences in extramural venous invasion (*t* =  − 2.113, *P* = 0.046). *K*^trans^ was strongly positively correlated with CD34 (*r* = 0.708, *P* = 0.000) and moderately positively correlated with vimentin (*r* = 0.450, *P* = 0.027). Interstitial volume (Ve) was moderately positively correlated with Masson’s (*r* = 0.548, *P* = 0.006) and vimentin (*r* = 0.417, *P* = 0.043). There was a moderate negative correlation between Ve and CDX-2 (*r* =  − 0.441, *P* = 0.031). The rate constant from extracellular extravascular space to blood plasma (Kep) showed a strong positive correlation with CD34 expression (*r* = 0.622, *P* = 0.001). ECV showed a moderate negative correlation with CDX-2 (*r* =  − 0.472, *P* = 0.020) and a moderate positive correlation with collagen fibers (*r* = 0.558, *P* = 0.005).

**Conclusion:**

The dynamic contrast-enhanced MRI-derived parameters measured in rectal cancer were significantly correlated with the proportion of histological components. This may serve as an optimal imaging biomarker to identify tumor tissue components.

## Introduction

Colorectal cancer (CRC) is the third most common cause of cancer worldwide, accounting for up to 9.3% of cancer-related deaths in 2020 [1]. Approximately 4.4% of males (1 in 23) and 4.1% of females (1 in 25) were diagnosed with CRC in their lifetime [2]. Rectal cancer accounts for one-third of all colorectal cancers [3]. Magnetic resonance imaging (MRI) has become a key diagnostic tool for rectal cancer because of its excellent soft-tissue contrast in complex and heterogeneous areas. MR imaging can provide information about T and N stages, extramural vascular invasion, and its relationship with surrounding structures [4].

Since the routine implementation of MRI for rectal cancer, advanced functional MRI sequences including diffusion-weighted imaging (DWI) and dynamic contrast enhancement (DCE) have demonstrated promising results in tumor heterogeneity, overcoming some limitations of conventional MRI in the evaluation of rectal cancer. DCE-MRI combines arterial input function and pharmacokinetic models to assess tissue perfusion, vasculature, capillary permeability, and interstitial space volume [5]. It can be used to assess tumor vascularization and help to determine the aggressiveness, angiogenesis degree, and staging of tumors [6]. A previous study found that the rate constant (Kep) values of the high-volume transfer constant (K^trans^) area in rectal cancer were positively correlated with microvessel density (MVD), and the whole transverse K^trans^ and Kep values of epidermal growth factor receptor (EGFR) expression positive group were higher [7]. DCE-MRI can also be used to monitor the response of rectal cancer to neoadjuvant chemoradiotherapy (CRT) [8]. Intven et al. found that both the post-CRT tumor volume and post-CRT K^trans^ values and the relative changes in volume and K^trans^ can predict pathological response, of which relative K^trans^ is the most predictive parameter [9]. However, the relationship between the pathological features of rectal cancer and the quantitative analysis of DCE-MRI is still unclear.

In this prospective study, we investigated the relationship among various tissue components (percentage of angiogenesis, nuclei of epithelial cells, nuclei of cancer cells, collagen fibers, and mesenchymal cells) and DCE-MR imaging parameters in vivo using whole-mount histological sections. The purpose of this study was to investigate the relationship between DCE-MR imaging measurements and the composition of rectal cancer.

## Methods

### Study population

**Fig. 1:**
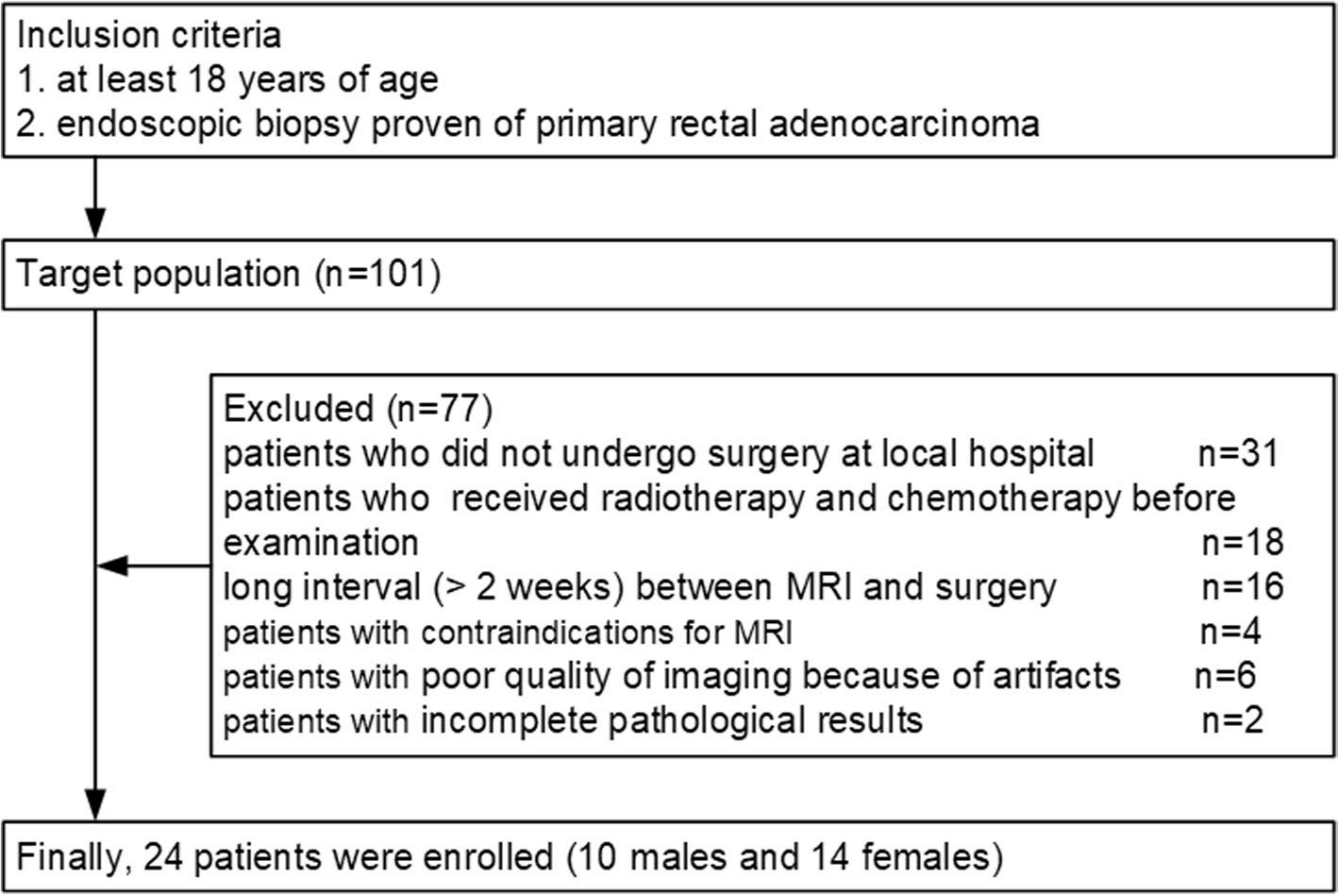
Flow diagram of the study population

From December 2019 to December 2020, DCE-MRI was performed before surgery in rectal cancer patients with endoscopic biopsy-proven primary rectal adenocarcinoma. Exclusion criteria were as follows: patients who did not undergo surgery at a local hospital (*n* = 31); patients who received radiotherapy and chemotherapy before examination (*n* = 18); long interval (> 2 weeks) between MRI and surgery (*n* = 16); patients with contraindications for MRI (*n* = 4); patients with poor quality of imaging because of motion or metal artifacts (*n* = 6); patients with incomplete pathological results (*n* = 2). The inclusion and exclusion criteria are shown in Fig. 1. Finally, 24 patients (median age, 65 years; age range, 35–82 years; 10 males and 14 females) who met these criteria were analyzed. The clinical data of the patients in this study are summarized in Table 1.

**Table 1:** Characteristics of 24 patients with rectal cancer

Characteristic	Value
Patient sex	
No. of male	10(41.7%)
No. of female	14(58.3%)
Age (years)	
All patients	64.79 ± 12.54 [35–82]
Male	62.50 ± 10.31 [41–76]
Female	66.43 ± 14.05 [35–82]
*P*	0.299
T stages	
pT2	3(12.5%)
pT3	21(87.5%)
pT3a	10(41.7%)
pT3b	5(20.8%)
pT3c	6(25.0%)
N stages	
pN0	11(45.8%)
pN1	7(29.2%)
pN2	6(25.0%)
Histologic grades	
G1	6(25.0%)
G2	12(50.0%)
G3	6(25.0%)

*T3a* tumor extends < 5 mm beyond muscularis propria, *T3b* tumor extends 5–10 mm beyond muscularis propria, *T3c* tumor extends 10 mm beyond muscularis propria

### MRI scan acquisition

All DCE-MR images were obtained using 3.0 T MRI (MAGNETOM Skyra, Siemens Healthcare, Erlangen, Germany) with an external dedicated 18-channel body coil. All subjects fasted for 4 h prior to scanning. Routine sequences included sagittal T2 weighted imaging (T2WI), axial T1WI, T2WI, DWI, and coronal T2WI. DCE-MRI acquisition used a T1 Twist Vibe sequence to scan the same axial layer as T2WI. Before the dynamic acquisition, an unenhanced T1 map based on a dual flip angle of 2° and 15° was obtained using the same sequence. A dose of 0.2 ml/kg body weight of gadolinium-diethylenetriamine pentacetate (Gd-DTPA) was injected intravenously at a rate of 2 ml/s using high-pressure syringes. Subsequently, 40 sets of contrast-enhanced images were acquired without delay. The detailed imaging parameters are as follows: temporal resolution, 7.4 s; total acquisition time, 5 min 3 s; TR/TE, 4.87/1.87 ms; flip angle, 12°; field of view, 200 mm; voxel size of 1.3 × 1.3 × 2.0 mm^3^; slice thickness, 2 mm; and slices per slab, 48. Subsequently, 20 ml of 0.90% NaCl saline solution was injected at the same rate.

### Image analysis

All DCE-MR images were post-processed using the software package PMI 0.4 (Platform for Research in Medicine Imaging), written in IDL 6.4. The tracer-kinetic modeling for the quantitation of DCE-MRI images was based on a modified Tofts pharmacokinetic model [10], as follows:

$${C}_{t}\left(t\right)={v}_{p}{C}_{p}\left(t\right)+{K}^{Trans}{\int }_{0}^{t}{C}_{p}\left(\tau \right) exp\left[-\frac{{K}^{Trans}(t-\tau )}{{v}_{e}}\right]d\tau$$

Kep equaled the ratio K^trans^/Ve.

The images were examined by a radiologist (J. Y.) specializing in MRI with 8 years of experience in abdominal MR imaging and a radiologist (YC. Y.) specializing in MRI with 11 years of experience in abdominal MR imaging. Both of them evaluated the images together to determine the regions of interest (ROIs). Differences were resolved in the presence of a third senior radiologist (WL. T.) with 15 years of experience in abdominal radiology. By comparing with T2WI images, ROIs were selected for manual delineation in the deepest plane of rectal cancer infiltration to avoid bleeding, necrotic cystic areas, intestinal contents, and the mesentery.

Arterial input function (AIF) was measured by manually drawing a small circular ROI on the lateral side of the iliac bone near the tumor.

Once the ROI was defined, the model-based parameters were measured, including volume transfer constant between the plasma and the extracellular extravascular space (K^trans^, in ml per 100 ml per min), rate constant from extracellular extravascular space to blood plasma (Kep, in ml per 100 ml per min), interstitial volume (Ve, in ml/100 ml), plasma volume (Vp, in ml/100 ml), mean transit time (MTT, in seconds), and extracellular volume (ECV, %).

### Surgical resection and histopathological staining

All surgical resections were performed by the same surgeon (HJ. G.), with 20 years of experience in gastrointestinal surgery. After surgery, freshly excised rectal specimens were immersed in formalin for at least 24 h. After formalin fixation, the surgically resected specimens were routinely sectioned on a plane perpendicular to the long axis of the intestine. A pathologist (K. L.) with 18 years of experience and a radiologist (J. Y.) with 8 years of experience referred to the T2W images and selected the slides with the deepest tumor infiltration for paraffin embedding. The tissues were processed using a standard histological procedure and stained with hematoxylin–eosin (HE). The standard immunohistochemistry (IHC) protocol was used to stain rectal cancer tissues using CD34 monoclonal antibody, caudal type homeobox transcription factor 2 (CDX-2) antibody, Ki-67 antibody, and vimentin antibody. Masson’s trichrome staining was performed using a ready-to-use kit.

### Histopathological evaluation

**Fig. 2:**
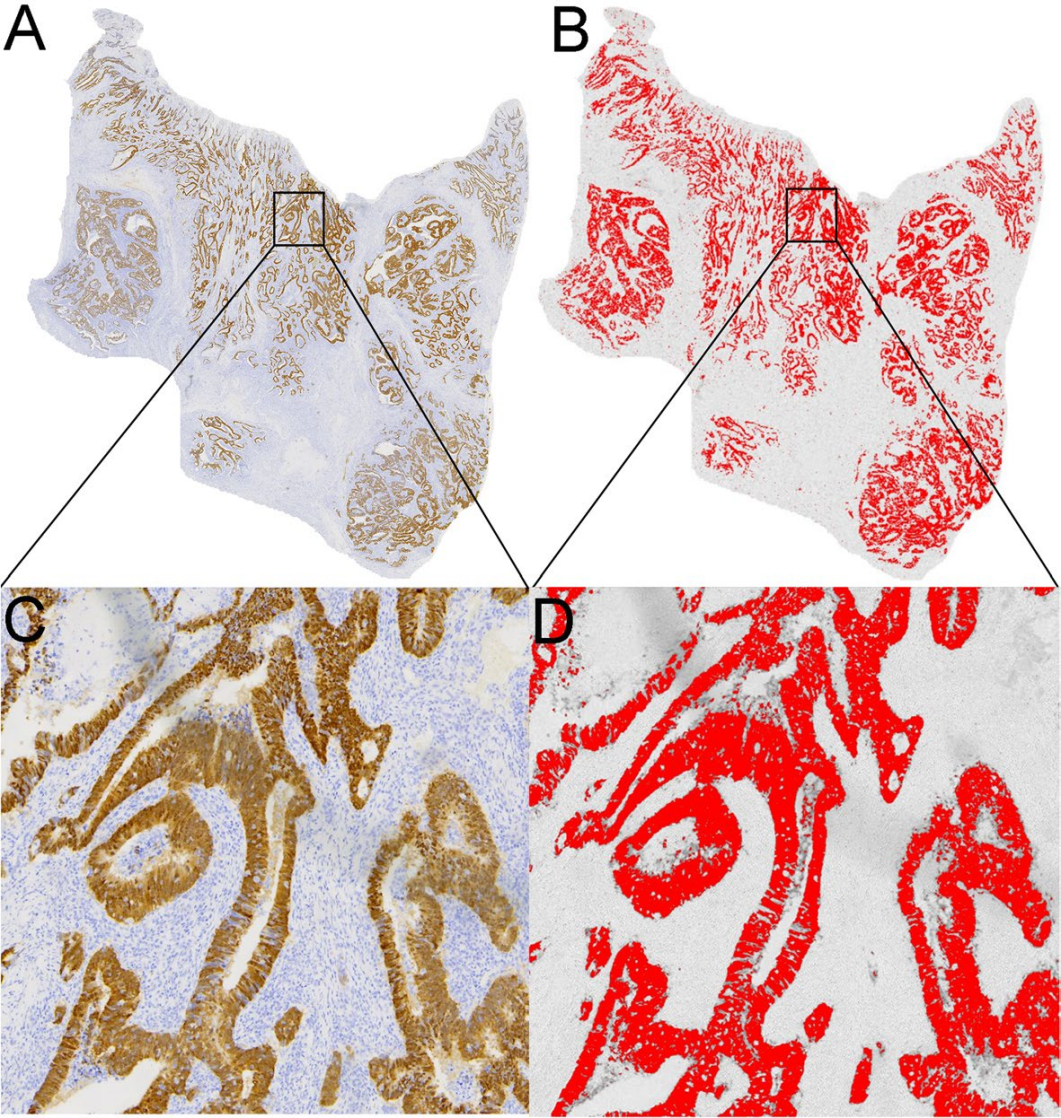
Representative images for quantitative histologic evaluation. **a**, **c** CDX-2 expression. Images were analyzed based on color selection. **b**, **d** The image analysis figure. The area labeled (in red) was calculated accordingly. The surface area was calculated as 20.74%

A full set of immunohistochemistry and Masson staining sections were generated and digitized at histological resolution. All slides were scanned using a MoticEasyScan scanner at × 20 magnification (pixel resolution 0.24 μm). The tumor areas were extracted from the whole-slide images by a pathologist with 18 years of experience. We analyzed the immunohistochemical expression areas (ratio; %) of CD34, CDX-2, Ki-67, vimentin staining (brown color), and Masson staining (bluish color) in tumors. The percentage of the tissue components was measured using image segmentation. The IHC profiler plugin with digital image analysis software ImageJ was used for image processing and measurement and was analyzed by Varghese et al. [11]. Using the “area method”, the total area occupied by positively stained areas and negatively stained areas can be selected using ImageJ’s threshold tool (Fig. 2). The positive IHC and Masson indices of the images were calculated.

### Statistical analysis

Statistical analyses were performed using IBM SPSS Statistics for Windows, version 20.0 (IBM Corp, Armonk, NY, USA). For each cancer, the mean DCE-MRI parameters and positive staining percentages of cancer components (CD34, CDX-2, Ki-67, vimentin, and collagen) were calculated. One-way analysis of variance (ANOVA) was used to test the differences in DCE-MRI parameters, and different differentiation types, different T stages, and N stages. An independent samples *t* test was used to test the differences between DCE-MRI parameters and venous and neural invasion. Data were normally distributed using the Kolmogorov–Smirnov test. Pearson correlation analysis was applied to evaluate the correlation between pathology parameters and DCE-MRI parameters. The person correlation coefficients were interpreted as follows: weak, 0.2–0.4; moderate, 0.4–0.6; and strong, > 0.6. Multiple linear regression analysis was used to analyze the histopathological parameters of DCE-MRI. The statistical significance was set at *P* < 0.05.

## Results

### Correlation between DCE-MRI parameters with cancer staging and grading

On the WHO grading scale, significant differences were observed in K^trans^ among G1(6), G2(12), G3(6) (*P* = 0.001), and MTT (*P* = 0.038), as shown in Table 2. There were no significant differences in VP (*P* = 0.955), Ve (*P* = 0.297), Kep (*P* = 0.134), and ECV (*P* = 0.544).

**Table 2:** Correlation between DCE-MRI parameters with cancer staging and grading

		Vp (ml/100 ml)	Ve (ml/100 ml)	MTT (s)	K^trans^ (ml/100 ml/min)	Kep (ml/100 ml/min)	ECV (%)
Histologic grades							
G1	6	4.552.35	20.21 ± 5.41	96.56 ± 62.94	14.01 ± 5.92	83.70 ± 42.79	24.92 ± 10.70
G2	12	4.80 ± 3.20	24.46 ± 8.11	51.23 ± 17.14	30.66 ± 9.93	131.77 ± 49.86	28.26 ± 8.17
G3	6	4.28 ± 4.64	27.88 ± 10.76	51.84 ± 19.77	32.87 ± 6.41	130.22 ± 48.37	30.79 ± 9.45
***F***		0.047	1.289	9.890	9.890	2.219	0.627
*P*		0.955	0.297	0.038	0.001	0.134	0.544
T stages							
T2	3	6.79 ± 2.10	29.45 ± 9.61	59.10 ± 14.32	30.68 ± 9.49	106.33 ± 29.80	34.85 ± 9.35
T3a	10	3.43 ± 2.31	24.70 ± 7.44	76.63 ± 54.33	25.40 ± 11.36	106.03 ± 48.12	26.80 ± 6.82
T3b	5	3.06 ± 3.80	25.44 ± 12.69	54.52 ± 23.15	29.40 ± 12.50	131.58 ± 68.28	27.81 ± 13.30
T3c	6	6.77 ± 3.64	19.94 ± 8.41	48.16 ± 17.09	26.02 ± 12.47	137.93 ± 49.04	26.96 ± 8.89
***F***		2.518	0.944	0.780	0.245	0.638	0.635
*P*		0.087	0.438	0.519	0.864	0.599	0.601
N stages							
N0	11	5.64 ± 2.74	23.39 ± 7.68	56.37 ± 14.87	25.90 ± 7.89	114.68 ± 34.62	27.83 ± 7.73
N1	7	2.33 ± 2.22	27.24 ± 12.13	88.91 ± 62.61	25.49 ± 15.21	99.62 ± 60.02	27.86 ± 12.37
N2	6	5.37 ± 4.31	22.37 ± 3.54	43.79 ± 14.37	30.97 ± 11.92	150.97 ± 56.27	28.72 ± 8.23
***F***		2.735	0.629	2.901	0.477	1.904	0.020
*P*		0.088	0.543	0.077	0.627	0.174	0.980
Venous invasion							
Negative	15	5.02 ± 3.80	21.98 ± 7.82	64.37 ± 48.18	26.94 ± 12.72	128.00 ± 59.38	25.26 ± 8.57
Positive	9	3.92 ± 2.23	28.06 ± 8.38	59.95 ± 13.94	27.22 ± 8.51	104.97 ± 27.48	32.72 ± 8.02
*t*		0.790	− 1.795	0.266	− 0.058	1.289	− 2.113
*P*		0.438	0.086	0.792	0.954	0.211	0.046
Nerve invasion							
Negative	17	5.29 ± 3.39	23.35 ± 9.15	67.05 ± 44.09	25.28 ± 10.57	113.93 ± 45.23	27.09 ± 9.15
Positive	7	2.95 ± 2.52	26.45 ± 6.32	52.20 ± 18.09	31.35 ± 12.08	132.55 ± 63.17	30.42 ± 8.79
*t*		1.638	− 0.816	0.853	− 1.228	− 0.816	− 0.819
*P*		0.116	0.423	0.403	0.233	0.423	0.422

There were no significant differences in VP (*P* = 0.087; *P* = 0.088;* P* = 0.116), Ve (*P* = 0.438; *P* = 0.543;* P* = 0.423), MTT (*P* = 0.519; *P* = 0.077;* P* = 0.403), K^trans^ (*P* = 0.864; *P* = 0.627;* P* = 0.233), Kep (*P* = 0.599; *P* = 0.174;* P* = 0.423), and ECV (*P* = 0.601; *P* = 0.980;* P* = 0.422) in the subgroups with different T stages, different N stages, and nerve invasions.

ECV showed significant differences in extramural venous invasion (*P* = 0.046). There were no significant differences in VP (*P* = 0.438), Ve (*P* = 0.086), MTT (*P* = 0.792), K^trans^ (*P* = 0.954), and Kep (*P* = 0.211).

### Correlations of histopathological findings with cancer staging and grading

On the WHO grading scale, significant differences were observed in CDX-2 and Ki-67 among G1, G2, and G3 (*F* = 4.935, *P* = 0.018; *F* = 4.131, *P* = 0.031). There were no significant differences in CD34 (*F* = 0.966, *P* = 0.397), collagen fibers (*F* = 1.258, *P* = 0.305), or vimentin (*F* = 2.129, *P* = 0.144). There were no significant differences in CD34 (*F* = 0.621, *P* = 0.609; *F* = 0.044, *P* = 0.957), CDX-2 (*F* = 0.779, *P* = 0.520; *F* = 0.210, *P* = 0.812), Ki-67 (*F* = 1.094, *P* = 0.375; *F* = 0.649, *P* = 0.533), collagen fibers (*F* = 0.199, *P* = 0.896; *F* = 1.000, *P* = 0.385), and vimentin (*F* = 1.155, *P* = 0.351; *F* = 1.412, *P* = 0.266) in subgroups with different T stages and N stages. There were no significant differences in CD34 (*t* = 0.745, *P* = 0.464; *t* = 0.179, *P* = 0.862), CDX-2 (*t* = 1.538, *P* = 0.138; *t* = 1.083, *P* = 0.290), Ki-67 (*t* = 1.050, *P* = 0.305; *t* = 1.054, *P* = 0.304), collagen fibers (*t* =  − 1.212, *P* = 0.238; *t* =  − 0.818, *P* = 0.422), and vimentin (*t* =  − 0.335, *P* = 0.740; *t* =  − 1.898, *P* = 0.071) in extramural venous invasion and nerve invasion.

### Correlation between DCE-MRI parameters and histopathological findings

**Fig. 3:**
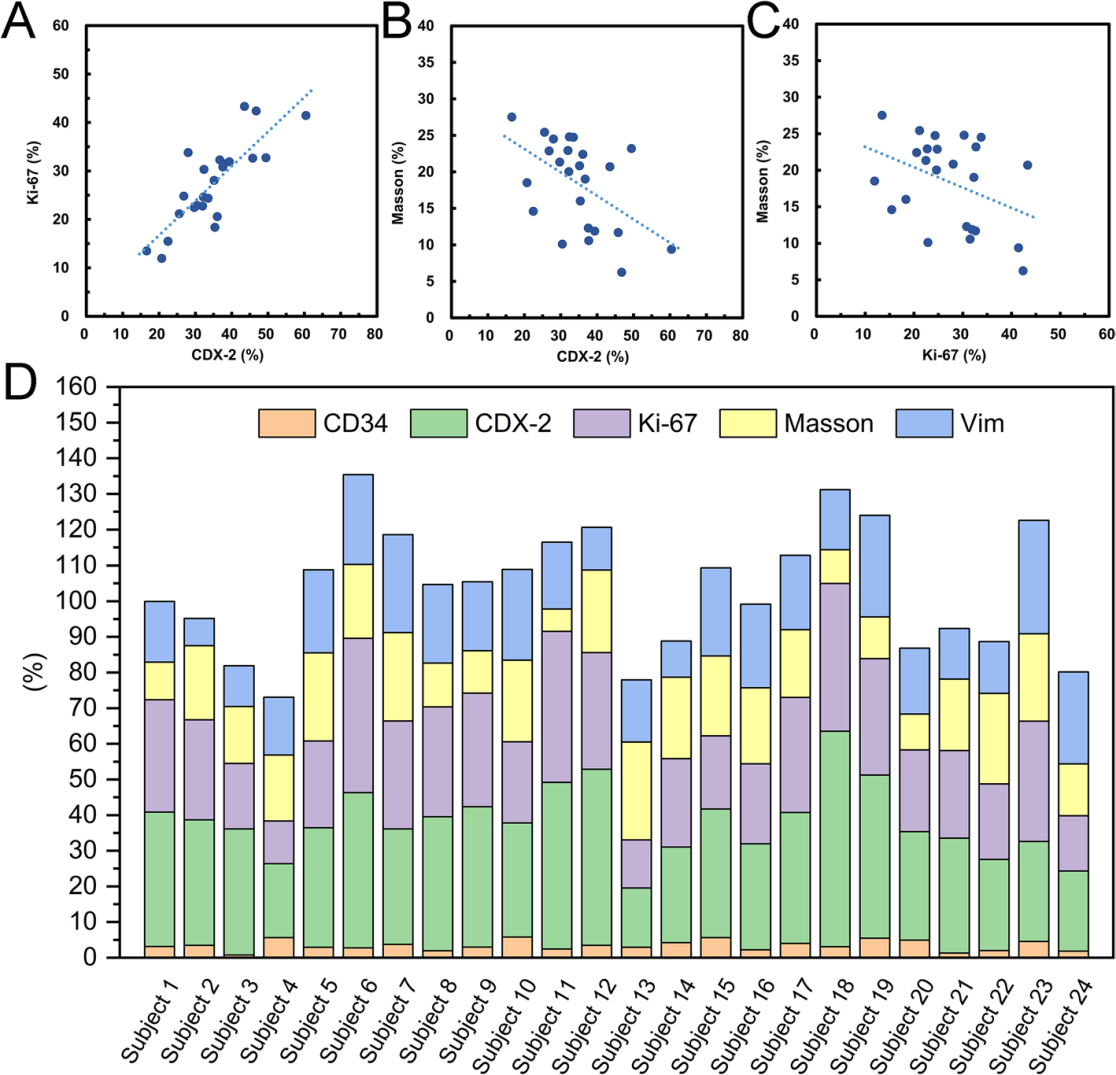
Graphics (**A**-**C**) show the correlations between histopathological markers (CDX-2, Ki-67, and Masson). Histograms show quantitative levels of histopathological markers (CD34, CDX-2, Ki-67, Masson’s, and vimentin) in 24 cases

There was a strong positive correlation between CDX-2 and Ki-67 levels (*r* = 0.814, *P* = 0.000), as shown in Fig. 3A. CDX-2 showed a moderate negative correlation with collagen fibers (*r* =  − 0.515, *P* = 0.010), as shown in Fig. 3B. Ki-67 showed a weak negative correlation with collagen fibers (*r* =  − 0.390, *P* = 0.060) as shown in Fig. 3C. The quantitative levels of CD34, CDX-2, Ki-67, vimentin, and Masson’s trichrome staining are shown in Fig. 3D.

**Fig. 4:**
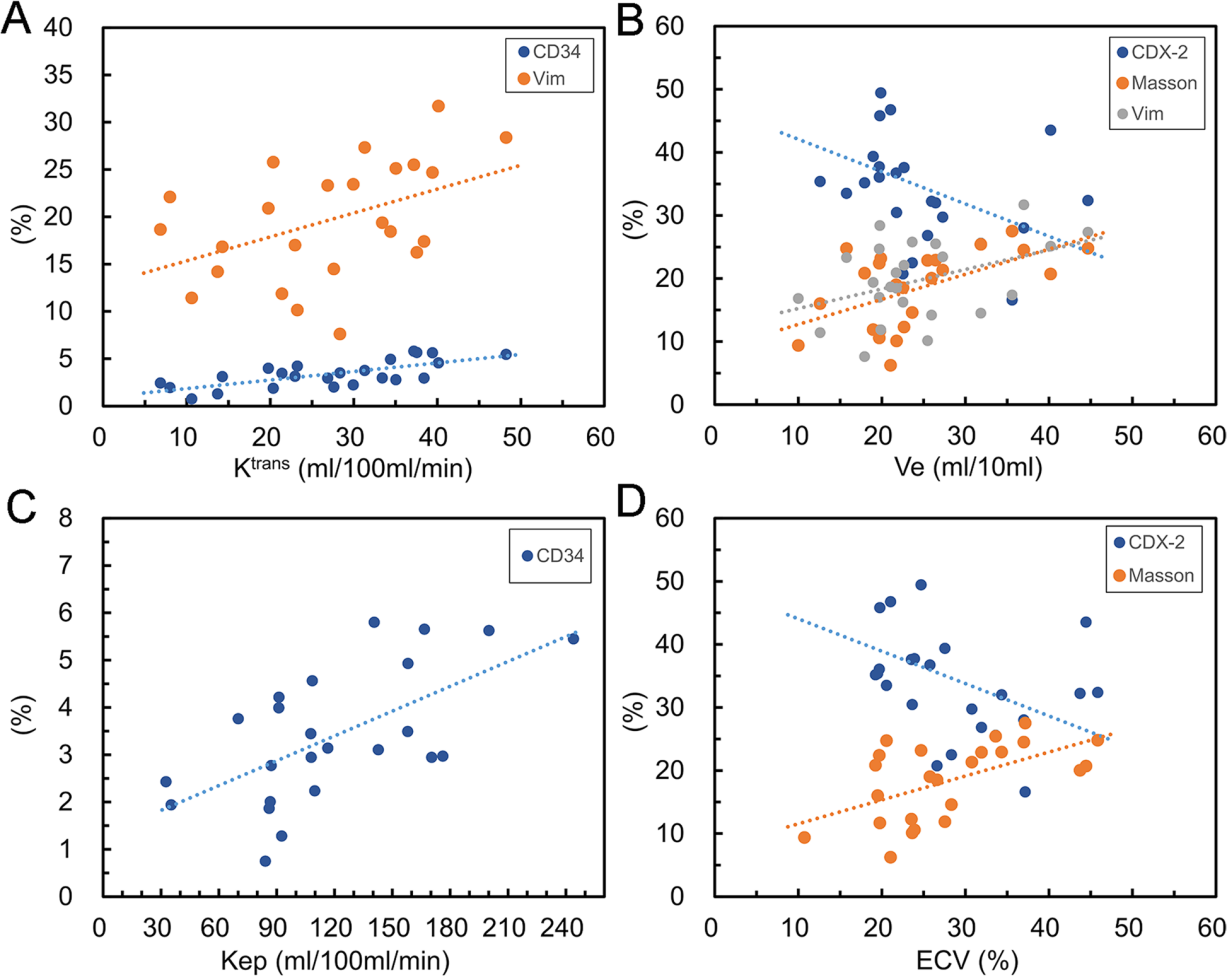
Correlations between DCE-MRI-derived parameters with histopathological markers in rectal cancer

**Fig. 5:**
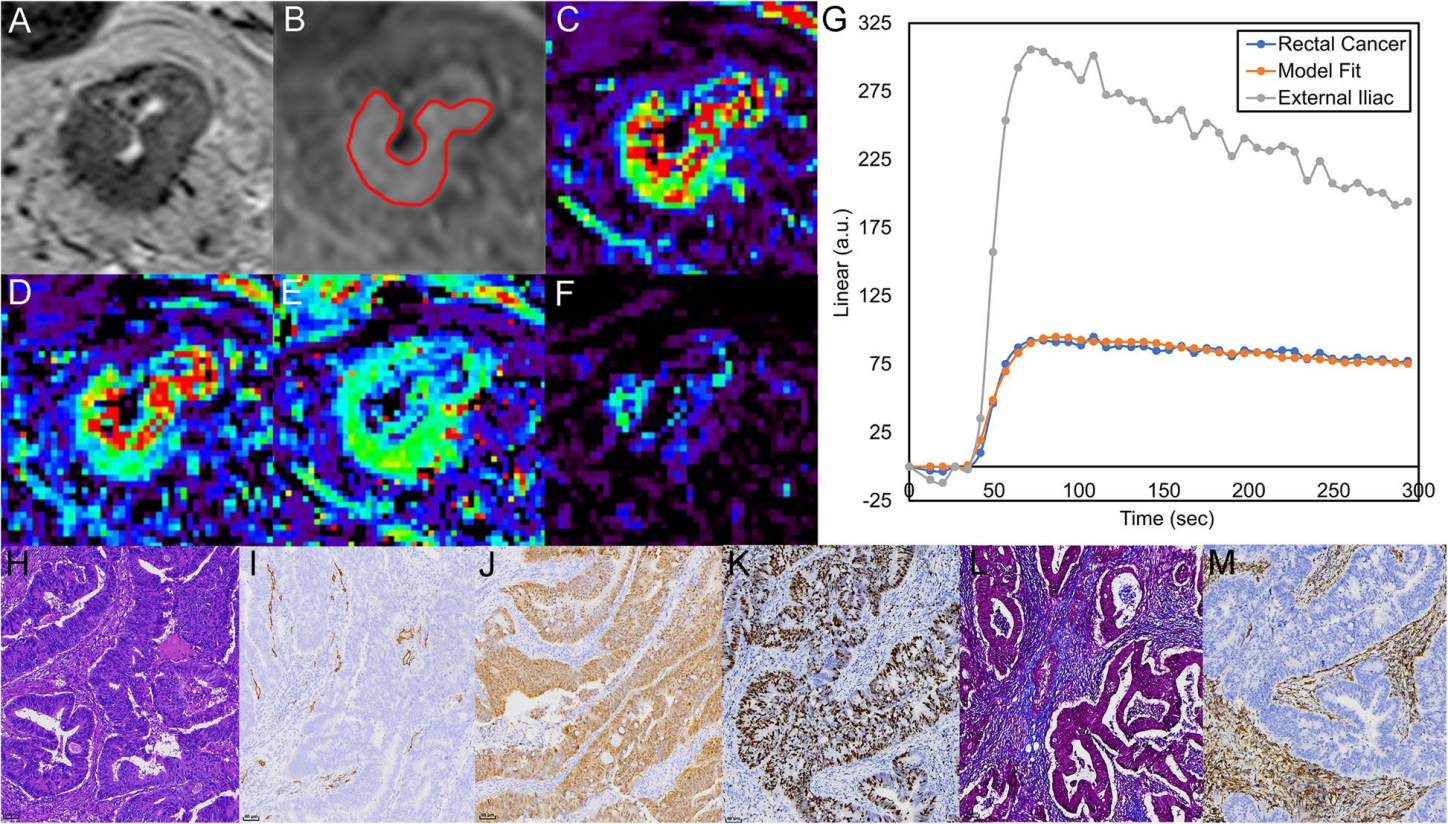
**a**, **b** Images of T2WI and DCE-MRI. **c**–**f** Corresponding parametric Ktrans, Kep, Ve, and Vp maps. The enhancement curve produced by a rectal adenocarcinoma (blue line), a model fit (orange line), and external iliac proximal (gray line) in g. a.u. = arbitrary units. **h**–**m** The images of HE, CD34, CDX-2, Ki-67, Masson’s, and vimentin staining

K^trans^ showed a strong positive correlation with CD34 (*r* = 0.708, *P* = 0.000) and a moderate positive correlation with vimentin (*r* = 0.450, *P* = 0.027). There was a weak positive correlation between K^trans^ and collagen fibers (*r* = 0.384, *P* = 0.064). K^trans^ showed a weak negative correlation with CDX-2 (*r* =  − 0.311, *P* = 0.139) and Ki-67 (*r* =  − 0.225, *P* = 0.290). Ve was moderately positively correlated with collagen fibers (*r* = 0.548, *P* = 0.006) and vimentin (*r* = 0.417, *P* = 0.043). A moderate negative correlation was observed between Ve and CDX-2 (*r* =  − 0.441, *P* = 0.031). Kep showed a strong positive correlation with CD34 expression (*r* = 0.622, *P* = 0.001). ECV showed a moderate negative correlation with CDX-2 (*r* =  − 0.472, *P* = 0.020) and a moderate positive correlation with collagen fibers (*r* = 0.558, *P* = 0.005). There was a weak positive correlation between ECV and vimentin (*r* = 0.260, *P* = 0.221), as shown in Figs. 4 and 5.

## Discussion

In the present study, we assessed the relationship between DCE-MRI measurements and rectal cancer tissue composition. The results revealed that DCE-MRI-derived parameters had significant correlations with the tumor tissue components in rectal adenocarcinoma. They may also be used to distinguish between different histological grades and extramural venous invasion.

CD34 is a transmembrane glycoprotein that exists in other types of stem/progenitor cells, including vascular endothelial progenitor cells [12]. Anti-CD34 monoclonal antibodies can recognize neovascularization in tumors. A previous study has shown that intratumoral neovascularization is an important independent predictor of tumor recurrence and recurrence time of colorectal cancer [13]. Therefore, evaluating intratumoral angiogenesis may help to better estimate individual survival and select patients with a higher risk of recurrence. At the same time, it can also be used to evaluate the efficacy of chemoradiotherapy and antivascular therapy. K^trans^ reflects the efflux rate of contrast agents from blood plasma into the extravascular extracellular space. Kep represents the transfer rate of contrast agents from the extravascular extracellular space to the blood vessels. They are related to capillary permeability and depend on the blood flow and capillary surface area. We found that both K^trans^ and Kep showed strong positive correlations with CD34 and that K^trans^ had a stronger correlation with CD34. Compared with normal tissues, tumors require more stringent nutrition and oxygen supply, as well as a greater ability to eliminate metabolic waste and carbon dioxide; therefore, they need to generate more new blood vessels [14]. Angiogenesis is the formation of new blood vessels from preexisting blood vessels, resulting in numerous, irregular, tortuous, fragile, hyperpermeable blood vessels. This makes it easy for blood to quickly leak out of the blood circulation and eventually leads to an increase in K^trans^ and allows blood to flow quickly into the blood vessels and eventually leads to an increase in Kep. Li et al. also found that K^trans^ and Kep were markedly higher in ductal carcinoma in situ and invasive ductal carcinoma than in mammary ductal dysplasia, and that K^trans^ and Kep values were positively correlated with CD105 levels [15]. In our study, K^trans^ and Kep derived from DCE-MRI can be used as imaging markers to predict the blood vessels in rectal cancer.

It is not difficult to understand that in our study, high-grade rectal cancer showed higher K^trans^ than low-grade rectal cancer. This is consistent with findings from studies of other malignancies. Jung et al. found that K^trans^ was the most significant parameter for distinguishing high- and low-grade gliomas [16]. Another study found mean K^trans^ is higher in breast cancer with high histologic than that in breast cancer with low histologic grade [17]. In this study, we did not find that Kep and Ve were related to the differentiation of rectal cancer. Our study supports that K^trans^ is the most important parameter for evaluating histological grades in rectal cancer. We believe that with the increase in the degree of rectal cancer differentiation, the reduction in the normal rectal wall layer, the loss of microcirculation, and the formation of neovascularization lead to an increase in K^trans^.

Malignant tumors include cancer cells and stroma. Both CDX-2 and Ki-67 showed nuclear staining in the tumors. Masson’s trichrome stain was used to stain collagen, which is an important component of the tumor stroma. CDX2 and Ki67 showed a strong positive correlation, while both CDX2 and Ki67 were significantly negatively correlated with collagen. These correlations demonstrate that cancer cells and stromal areas are interdependent. CDX-2 is expressed in colorectal cancer, plays an important role in the proliferation and differentiation of epithelial cells, and is used as a diagnostic marker. In a previous study, CDX-2 downregulation and deletion were significantly correlated with poor differentiation grade of CRC [18]. Another study found no correlation between CRC differentiation and CDX-2 expression in carcinomas, but the percentage of CDX-2 expression was generally lower than that in adenomas [19]. In our study, we found that CDX-2 was expressed in 100% of all rectal cancers, and the expression of CDX-2 in moderately and poorly differentiated carcinomas was significantly lower than that in well-differentiated carcinomas. This is because CDX-2 plays a role in the proliferation and differentiation of CRC cell, and the downregulation of CDX-2 expression may lead to the loss of tumor differentiation. Ki-67 is a nuclear nonhistone protein whose expression level can reflect the proliferative activity of tumors. High Ki-67 expression is associated with poor prognosis in many other tumor types, including head and neck, prostate, and breast cancer [20, 21]. However, we found that the expression was the highest in well-differentiated rectal cancer and the lowest in moderately differentiated tumors. Melling et al. found that high Ki-67 expression in CRCs was associated with good clinical outcomes [22]. Duchrow et al. suggested that large numbers of non-cycling tumor cells express Ki-67 in at least a third of CRCs; therefore, these tumors may grow more slowly than indicated by the Ki67 labeling index. These Ki-67 positive non-cycling tumor cells may be more stable than tumor cells that fail to achieve cell cycle arrest [23].

Collagen is the main component of the tumor stroma and is involved in tumor fibrosis. The overexpression of collagen in the tumor extracellular stroma is the main reason for the increase in tumor hardness in colorectal cancer. The hardness of colorectal cancer is one of the causes of mechanical stimulation, which is related to tumor invasion and metastasis, immune escape, and drug resistance [24]. ECV fraction derived from DCE-MRI represents extracellular volume fraction. ECV is recognized as a useful imaging biomarker for predicting treatment response and survival in patients with pancreatic ductal adenocarcinoma after chemotherapy [25]. Collagen expressed by Masson’s staining is an important component of the tumor stroma, and cancer cells expressed by CDX-2 staining are an important component of the tumor body. In our study, ECV was moderately positively correlated with collagen and moderately negatively correlated with CDX-2 expression. Therefore, it is not difficult to understand the correlation between ECV and CDX-2 and Masson staining. We also found a moderately positive correlation between Ve and collagen. Ve represents the volume of the extravascular extracellular leakage space, which is determined by the fractional distribution volume of the contrast agent in the tumor tissue. Klaassen et al. found a significant positive correlation between collagen fraction and Ve derived from DCE-MRI in pancreas cancer [26]. In this study, ECV was significantly higher in tumors with extramural venous invasion than that in tumors without extramural venous invasion. Nishishita et al. found that the extensive-expression of cancer-associated fibroblast markers, including collagen I was significantly correlated with high-grade venous invasion [27]. We believe that the enlargement of ECV in rectal cancer may be caused by the extracellular matrix including collagen. The increase in collagen may lead to increased fluid pressure within the tumor, which may lead to vascular invasion.

Vimentin is one of the most widely expressed intermediate filament proteins in mesenchymal cells, including interstitial collagen, fibronectin, elastin, glycosaminoglycans, and a variety of cell types [28]. In our study, vimentin expression is abundant in rectal cancer stroma, although rectal cancer cells did not express vimentin. It showed a significant positive correlation with Ve. The Increased stromal vimentin expression is thought to reflect the dynamic changes of tumor stroma during progression. It is an epithelial-mesenchymal transition (EMT)-associated marker that is known to be correlated with progression, chemosensitivity, and metastasis in colorectal cancer [29]. Ngan et al. found that vimentin expression in tumor stroma can reflect higher malignant potential and is a useful predictive marker of colorectal cancer disease recurrence [30]. A previous study has suggested that positive vimentin immunostaining is associated with high-grade colorectal cancer [31]. However, our study did not find a significant correlation between vimentin expression and rectal cancer grade. In this study, vimentin expression positively correlated with K^trans^. Our study suggests that vimentin is related to tumor prognosis, possibly because it is related to the permeability of tumor neovascularization.

In our study, the MTT assay showed significant differences in tumor grade. MTT was lower in G2 and G3 tumors than that in G1 tumors. The MTT represents the average time required for blood to travel from the arterial end to the venous end. Compared with G1, the decrease in MTT in G2 and G3 may have been caused by the opening of arterio-venous shunts in the tumor. The pathological junctions between arteries and veins lead to decreased blood flow resistance and increased blood flow between the pathological microvessels [32]. This results in a decrease in vascular MTT.

In this pilot study, we used immunohistochemistry and special staining to identify the components of tumor tissues and explore their correlation with DCE-MRI parameters. This study excluded patients with radiotherapy and chemotherapy mainly to understand the relationship between various components in rectal cancer tumors without radiotherapy and chemotherapy. To provide a basis for future evaluation of tumor composition after treatment. In future studies, we will investigate the changes of imaging markers in patients treated with radiotherapy and chemotherapy to infer changes in tumor composition. There are also some limitations to this study. (1) It is a pilot study with a small sample size that will require future evaluation in a larger patient population. (2) Previous studies have explored the correlations between tissue microanatomy in prostate cancer and laryngeal and hypopharyngeal carcinoma by HE staining of whole tumor sections with MRI-derived parameters [33, 34]. Our study confirmed the correlations between DCE-MRI parameters and tumor interior by immunohistochemical staining and Masson’s Trichrome staining. However, compared with previous studies, although we selected an image plane of the most infiltrated layer of the tumor to draw ROIs and analyze the immunohistochemical expression areas in whole-mount slides, they may not be exactly the same. To overcome this limitation, it is necessary to precisely locate and analyze the whole-tumor specimen to accurately reflect the characteristics of the whole tumor. In addition, follow-up studies will study as many cases as possible to improve the accuracy of research data and provide better information. (3) In addition, although CD34 has high sensitivity and specificity for vascular recognition, it cannot distinguish endothelial cells from normal vessels and tumor neovascularization. In future studies, more specific tumor neovascularization markers should be used for quantification.

## Conclusion

The dynamic contrast-enhanced MRI-derived parameters measured in rectal cancer were significantly related to the proportion of histological components. This may potentially serve as an optimal imaging biomarker for identifying tumor tissue components, monitoring therapy, or surveillance.

## Data Availability

Due to restrictions on ethical approval involving patient data and anonymity, the datasets analyzed during the current study are not publicly available but can be obtained from the appropriate authors upon reasonable request. If you would like to obtain data from the study, please contact corresponding author Professor Songhua Zhan.
